# Efficacy of Routine Fecal Microbiota Transplantation for Treatment of Recurrent *Clostridium difficile* Infection: A Retrospective Cohort Study

**DOI:** 10.1155/2019/7395127

**Published:** 2019-07-01

**Authors:** Alexandra Nowak, Magnus Hedenstierna, Johan Ursing, Christer Lidman, Piotr Nowak

**Affiliations:** ^1^Department of Nephrology, Karolinska University Hospital Huddinge, Huddinge, Sweden; ^2^Department of Infectious Diseases, Danderyds Hospital, Danderyd, Sweden; ^3^Department of Clinical Sciences, Danderyds Hospital, Danderyd, Sweden; ^4^Department of Medicin Huddinge, Unit of Infectious Diseases, Karolinska Institutet, Karolinska University Hospital, Huddinge, Sweden

## Abstract

**Background:**

Patients with recurrent *Clostridium difficile* infections (CDIs) constitute an increasing treatment problem. Fecal microbiota transplantation (FMT) has shown promising results of treating recurrent CDI, where treatment with antibiotics fails repeatedly. Our study describes retrospective cohort treated with FMT at two major hospitals in Stockholm.

**Methods:**

Medical records of all patients with recurrent CDI treated with FMT during the period 2013–2017 were reviewed. We evaluated cure of CDI-related diarrhea without relapse 10 weeks after FMT.

**Results:**

47 patients were included. One treatment cured 25 patients (53%), and more than one treatment cured 32 patients (68%). Treatment outcome did not vary significantly with treatment with fresh donor feces or frozen fecal culture, days of use of antibiotics or days of hospitalization prior to CDI, and renal function or time from the first CDI to therapy. Treatment failure was associated with a significantly lower Karnofsky performance status score (70 points vs 90, *p*=0.02).

**Conclusion:**

Fecal instillation, for the treatment of relapsing CDI, is a promising approach, with 68% success rate reported in this study. The success rate of FMT is high, regardless of multiple comorbidities, extended use of antibiotics, or long time hospitalization. Although generally FMT is performed with fresh donor feces, our data show that the usage of frozen fecal culture could be an effective treatment alternative in recurrent CDI.

## 1. Introduction

*Clostridium difficile* is an anaerobic Gram-positive, spore-forming, toxin-producing bacillus that is transmitted among humans through the fecal-oral route. *C. difficile* spores resistant to heat, acid, and antibiotics are common in health care facilities and low levels are found in the environment and food, allowing for nosocomial and community transmission [1].

In the United States, *C. difficile* is the most frequently reported nosocomial pathogen. A surveillance study in 2011 identified 453,000 cases of cases of *C. difficile infection* (CDI) and 29,000 deaths associated with CDI [1]. The Public Health Agency of Sweden estimates that there are approximately 400 cases of recurrent CDI/year in Stockholm area (almost 1100 cases/year of CDI in total) [2]. Infection is prevented by barrier properties of the fecal microbiota. Weakening of this defense by antibiotic treatment is the major risk factor for symptomatic *C. difficile* infection. Additionally, advanced age, use of proton pump inhibitors (PPI), and multiple comorbidities such as chronic heart and lung diseases, kidney failure, and diabetes also contribute to CDI susceptibility. However, symptoms of colitis do not develop in all colonized persons [1, 3].

According to the European Society of Clinical Microbiology and Infectious Diseases guidelines, first-line treatment of CDI includes either metronidazole or vancomycin [4]. CDI relapses in approximately 15–26% of patients treated for a first episode [5]. Treatment of a second or later recurrence commonly includes tapered or long-term vancomycin but is even less effective with over 50% risk for continued recurrence [5, 6]. Due to high recurrence rates and alteration of colonic microbiota with the continued use of antimicrobial drugs, new approaches in CDI therapy include new narrow-spectrum antibiotics, probiotics, and monoclonal antibodies [7, 8]. For example, fidaxomicin has been shown to reduce the rate of recurrence compared with vancomycin; however, its use is limited by costs [9].

A promising way of treating recurrent CDI, which has attracted much interest in recent years, is reintroduction of normal intestinal flora by infusion of feces from healthy donors or a stool substitute obtained from purified fecal cultures [10]. Restoring normal colon microbiota was first described by Eiseman and coworkers in 1958 [11]. Reports from case series and meta-analyses show success rates from 64–95% [12–15].

Fecal instillation with donated feces or purified feces culture has been used to routinely treat patients with multiple CDI recurrences at the Departments of Infectious Diseases at Danderyd Hospital and Karolinska University Hospital Huddinge in Stockholm/Sweden for many years. However, FMT has been reserved to treat the most difficult cases. This work presents the results of all 47 patients with recurrent CDI, treated with fecal instillation in the period 2013–2017.

## 2. Materials and Methods

### 2.1. Study Design

This was a retrospective analysis of the characteristics and outcomes of all patients with recurrent CDI treated in the period from September 2013 to September 2017.

### 2.2. Study Sites

The study was conducted at the departments of infectious diseases at Danderyd Hospital and Karolinska University Hospital, Huddinge. The sites are two of Stockholm's major hospitals where FMT is done.

### 2.3. Data Collection

We examined electronic medical records for patients using a predefined data entry form that included information on clinical outcome 10 weeks after FMT. We also collected data on hospitalization 90 days prior to CDI and antimicrobial drug treatment 90 days prior to CDI.

Comorbidities were analyzed using the Charlson Comorbidity Index [16] which predicts the one-year mortality for a patient who may have a range of comorbid conditions such as heart and lung disease, dysfunction of the liver and kidney, and prior cerebral-vascular events diabetes or cancer (a total of 22 conditions). Each condition was assigned a score of 1, 2, 3, or 6, depending on the mortality risk associated with each one. Scores were summed to provide a total score to predict mortality. We also evaluated the Karnofsky Performance Scale Index [17], an estimate of the patient's overall performance status or ability to perform their activities of daily living. It is a single score between 10 and 100 assigned by a clinician based on observations of a patient's ability to perform common tasks relating to activity, work, and self-care. A score of 100 signifies normal physical abilities with no evidence of disease. Decreasing numbers indicate a reduced performance. In the study, we also recorded the use of proton pump inhibitors (PPI), renal function at CDI diagnosis, and days from first CDI to FMT. Additionally, we have collected the data on number of days to CDI recurrence, adverse events up to 10 weeks after FMT, and mortality 12 months after FMT.

### 2.4. Definitions

CDI was defined as the presence of unformed frequent stools (at least 3/day) for the last 48 hours and a positive PCR test for *C. difficile toxin* B DNA (Karolinska Microbiology Laboratory). Treatment success was defined as clinical cure 10 weeks after FMT [18, 19]. Individuals with a positive stool sample for *C. difficile* toxin B DNA during follow-up but no diarrhea were classified as a treatment success. Treatment failure was defined as recurrent diarrhea where the physician in charge decided to treat the patient for suspected or confirmed *C. difficile*. Adverse events were elicited by us for a modification of the Common Terminology Criteria for Adverse Events version 4.0 [20].

### 2.5. Feces Handling

Donors were healthy relatives or close contacts (without chronic diseases, diarrhea, and antibiotics usage within last two months). They were screened with serology for HIV, HBV, and HCV and by culturing of fecal samples and or PCRs for resistant *Enterococcus* (VRE), extended-spectrum beta lactamase (ESBL), *C. difficile*, *Salmonella, Shigella*, and *Campylobacte*r. Donor feces were delivered to the hospital within 24 hours of evacuation in a clean, closed plastic container. The donor feces were refrigerated until instillation, at the day of transplant. Approximately two table spoons of donor feces (corresponding to 30 g of feces) were diluted with 500 ml room temperate 0, 9% sodium chloride solution, and blended into a homogenous liquid in an electric blender.

The feces culture originates from 1994 and was obtained from a feces sample of healthy Scandinavian donor on ordinary Western diet as previously described [21]. The culture (30 ml) was stored at −70°C at the Department of Microbiology, Tumor and Cell biology Karolinska Institute and delivered to the hospital the day before transplant. The culture was thawed in room temperature 1 h prior to transplant.

### 2.6. Fecal Microbiota Transplantation Procedure

Patients were requested to cease treatment with vancomycin or metronidazole at least 24 h prior to instillation procedure. All patients received loperamide 1 h prior to the procedure to promote retention of the FMT. Rectal administration was performed in most of the patients (42 out of 47) via the rectal catheter during 30 min with the patient recumbent on the left side. In five cases, FMT was installed through a nasogastric tube. Patients were bedbound 1 h after the procedure and informed not to eat two hours after instillation.

### 2.7. Ethical Approval

Regional Ethics Committee Stockholm has reviewed and approved the study (dnr 2018/54-31/2).

### 2.8. Data Management and Statistical Analysis

The data are presented as median (range) as indicated. Nonparametric statistical analyses were applied with the Fisher exact test for the categorical and Mann–Whitney test for continuous variables, respectively. A *p* value <0.05 was considered as significant. All statistical analyses were performed in Graph-Pad Prism v 6.0.

## 3. Results

### 3.1. Study Population

During the study period (September 24, 2013, to September 7, 2017), a total of 48 patients with CDI were treated with FMT at Danderyd Hospital (*n*=28) and Karolinska University Hospital, Huddinge (*n*=20). One patient was excluded from the study due to lack of follow-up 10 weeks after FMT as the patient died due to severe lung fibrosis, not related to FMT (3 weeks after FMT). We have thus analyzed 47 patient records during the study period.

Thirty-three (62%) patients were women. Median age of the cohort was 70 years (range 25–95 years). Thirty-four patients had at least three recurrent CDI episodes prior to FMT. All patients had failed prior vancomycin tapering therapy. Of the 47 patients included in the study, eight patients (17%) had no use of antibiotics 90 days prior to the first episode of CDI and 12 patients (26%) were prescribed one antibiotic regime 90 days prior to CDI (Table 1).

Thirty-three (70%) patients received donor feces, and 14 (30%) patients received feces culture. There were no major adverse effects reported due to FMT. However, seven patients reported abdominal pain and/or flatulence during or immediately after FMT. One patient was admitted to hospital two days after FMT due to high fever and worsening of symptoms related to CDI. Three patients (6%) died during the 12-month follow-up period due to non-CDI/FMT-related causes.

### 3.2. Effects of FMT

**Figure 1:**
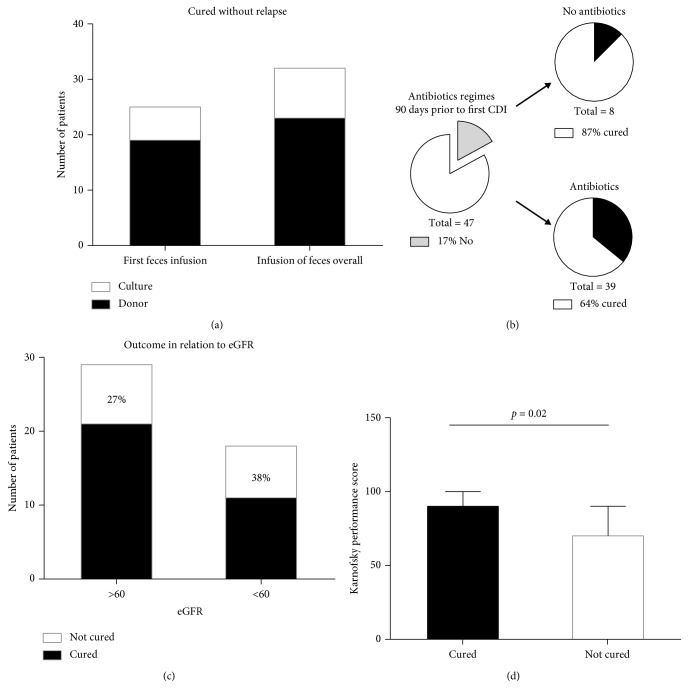
Outcome of fecal microbial transplantation in the cohort in relation to several factors: number of FMT treatments with donor feces or fecal culture (a); intake of antibiotics 90 days prior to *C. difficile* infection (b); eGFR (c); Karnofsky performance score (d).

Twenty-five patients (53%) were cured after one treatment. Seven additional patients were cured after 2–4 FMTs (Figure 1(a)), resulting in an overall cure rate of 68%. No significant difference was found between male and female patients, with cure rates of 12 (86%) of male and 20 (60%) of female patients, respectively.

In total, 23 (72%) patients who received donor feces and nine (64%) patients who received fecal culture were cured. Nineteen patients (57%) who received donor feces and six patients (43%) who received bacterial culture were cured after first FMT. CDI relapsed within three weeks after the procedure (median 7 days, range 1–45 days) in majority of patients in whom FMT failed (86%). The statistical analyses did not reveal any significant associations between cure rate and age, gender, or mode of FMT (donor vs cultures).

Seven of eight patients (87%) who had not been prescribed antibiotics 90 days prior to the first CDI were cured after FMT and 25 out of 39 patients (64%) who had been prescribed one or more antibiotic regimes within 90 days prior to CDI were cured (Figure 1(b)). The FMT cure rate was 60 and 65% in patients on short (1–10 days) and long (>11 days) antibiotic treatment (within 90 days prior to CDI), respectively.

The Charlson Comorbidity Index in the cohort was the same in the cured and non-cured groups (2 range 0–7), indicating a one-year mortality of 10%. Prior to FMT, 18 patients (38%) had an eGFR < 60. However, impaired renal function was not associated with the effectiveness of FMT (Figure 1(c)). The median Karnofsky performance score was significantly higher in the cured group compared to patients who had failed the FMT (median; 90 vs 70; *p* = 0.02) (Figure 1(d)). There was no difference in cure rate with regards to numbers of days to first FMT, number of comorbidities, GI surgery, PPI usage, and hospitalization 90 days prior to CDI.

## 4. Discussion

This retrospective study summarizes the outcome of FMT in 47 patients with recurrent CDI in routine clinical settings. FMT was a well-tolerated and effective treatment for CDI, with an overall success rate of 68%. Additionally, we report equal FMT efficacy using fresh donor stools as compared to frozen fecal cultures. The study highlights that FMT has a higher success rate in patients with a higher Karnofsky performance score indicating a generally healthier individual. Despite that, no association between cure rate and several factors previously described as associated with CDI treatment failure such as number of comorbidities, renal failure, days to FMT, and duration of antibiotics treatment was found. [1, 3] The high treatment success rate and relatively small number of patients may account for this lack of association. Nevertheless, an important overall observation in our study is that FMT has a high success rate regardless of age and comorbidities.

Higher FMT efficacy has been described in some but not all earlier studies [5, 10, 12, 21]. For instance, several studies reported overall cure rate of 80–95% [13, 22], while others showed success rates of 64–69% [12, 23], i.e., similar to the results presented here. We could not identify differences in baseline demographic or clinical characteristics of the patients in our study and the studies with higher FMT success rate. The difference in cure rate may depend on the dosage of fecal material as well as FMT procedures which are not standardized throughout studies. A single dose of FMT typically uses 50 g of fecal material, but a different amount of feces have been used ranging from 10–100 g of fecal material per dose [23–25]. According to the FMT protocol routinely applied in two study centers in Stockholm, approximately 30 g of fecal material was administered which may have affected the outcome in some patients. We cannot explain the lower cure rate with the choice of administrated material as we found no difference in the outcome of FMT regarding usage of fresh donor feces or frozen fecal cultures. Even if not fully applicable to our situation, a recent meta-analysis by Tang et al. [26] also indicates that the treatment efficacy of frozen FMT and fresh FMT was similar. A possible contributing factor to the lower efficacy might be selection of the most difficult to cure patients in this study as FMT in our setting was reserved for this patient group.

Additionally, some reports suggest that patients with recurrent CDI may benefit from multiple serial FMT procedures [14, 15, 27]. In the two Stockholm centers, single FMT procedures were routinely conducted, and in our study, we observed 10% increase of cure rate with additional FMT. However, as highlighted by Kelly and Tebas [28], much work remains to be done in defining the optimal FMT dose, recipient preparation, and post-FMT monitoring. In light of this, it is worthwhile noting that the fecal culture used in this study appeared to be as effective as freshly donated fecal material. This suggests that it is possible to establish one or perhaps several defined microbiota cultures that may enable standardized FMT in addition to having significant practical and regulatory implications.

Of the 47 patients included in the study, eight patients (17%) had no use of antibiotics 90 days prior to the first episode of CDI and 12 patients (26%) were prescribed one antibiotic regime 90 days prior to CDI. This illustrates that CDI in our cohort was not solely affecting individuals with repeated and extended antibiotic treatment and that a significant amount of CDI is community acquired as previously described [1].

Recovery of the intestinal flora is believed to be behind cure with FMT but the mechanism of FMT effect is still not fully resolved. It remains to be understood whether reconstituting the bacterial community alone or support from other microbes and/or metabolites play a role in the resolution [25]. Nevertheless, FMT is emerging as an effective therapy for recurrent CDI, and it is being investigated as a treatment option for other inflammatory conditions. More recently, clinical trials have focused on the role of FMT in inflammatory bowel disease (IBD) [29]. Whether FMT aids IBD control or provokes IBD exacerbation is an area of active debate, and at present, no conclusive statement can be made on the balance between FMT's benefits and risks in this population. Published case reports have also demonstrated clinical improvement after FMT in conditions not classically associated with gastrointestinal diseases such as multiple sclerosis, Parkinson's disease, alopecia areata, and idiopathic thrombocytopenic purpura [30–32]. This suggests not only a local intestinal effect but a profound immunological response to FMT. As such, more research is needed to explain mechanism of FMT and its immunological effects. Additionally, the long-term effects of microbiota manipulation are still unclear, especially in light of the increasingly recognized roles of the human gut microbiome in health and disease [33].

Knowing its limitations, we have to bear in mind that the FMT has been used for decades and has a definite place in treatment of the recurrent CDI. The results from our retrospective study in clinical settings fully support the future use of FMT in this patients group as safe procedure to cure CDI regardless of patients' comorbidities. Although generally the FMT is performed using the fresh or frozen donor stools, we propose that the usage of frozen fecal culture may be a fully effective alternative in recurrent CDI.

## Figures and Tables

**Table 1:** Clinical characteristics of the patients.

	All	Success	Failure	*p* value
Number of patients	47	32	15	—

Age				
Median, years (range)	70 (25–95)	69.5 (25–90)	74 (40–95)	n.s.

Gender				
Female, *n* (%)	33 (70)	20 (61)	13 (39)	n.s.
Male, *n* (%)	14 (30)	12 (86)	2 (14)	

Recurrent CDI, *n* (%)				
1-2	13 (27)	9 (28)	4 (26)	n.s.
3 or more	34 (73)	23 (72)	11 (74)	

Antibiotics regimes 90 days prior to the first CDI				
No, *n* (%)	8 (17)	7 (87.5)	1 (12.5)	n.s.
Yes, *n* (%)	39 (83)	25 (64)	14 (36)	

Days of antibiotics 90 days prior to CDI^*∗*^				
0 (%)	8 (17)	7 (87.5)	1 (12.5)	n.s.
1–10 (%)	15 (33)	9 (60)	6 (40)	
>11–20 (%)	23 (50)	15 (65)	8 (35)	

Reported use of PPI at CDI diagnosis (%)				
Yes	25 (53)	16 (64)	9 (36)	n.s.
No	22 (47)	16 (73)	6 (27)	

Days of hospitalization 90 days prior to CDI^*∗*^				
0 (%)	24 (52)	17 (71)	7 (29)	—
1–10 (%)	10 (22)	7 (70)	3 (30)	
>11–20 (%)	12 (26)	7 (58)	5 (42)	

Known GI surgery prior to CDI (%)				
Yes	16 (34)	13 (81)	3 (19)	—
No	31 (66)	19 (61)	12 (39)	

Kidney function at CDI (eGFR), *n* (%)				
>90	10 (22)	9 (90)	1 (10)	—
60–89	19 (40)	12 (63)	7 (37)	
HD and PD < 15–59	18 (38)	11 (61)	7 (39)	

Feces type (%)				
Culture no.	14 (30)	9 (64)	5 (36)	—
Donor no.	33 (70)	23 (70)	10 (30)	

Karnofsky performance status				
Median (range)	80 (40–100)	90 (50–100)	70 (40–100)	*p*=0.02

No. of comorbidities (%)				
0-1	14 (30)	11 (78)	3 (21)	—
2-3	23 (49)	14 (61)	9 (39)	
>4	10 (21)	7 (70)	3 (30)	

Days to first FMT				
Median	144	155.5	138	—
Range	9–884	9–884	40–337	

^*∗*^Data based on 46 of 47 individuals; *p* value <0.05 was considered as significant; n.s.: not significant.

## Data Availability

The clinical data used to support the findings of this study are available from the corresponding author upon request.
